# A bioactive peptide analogue for myxoma virus protein with a targeted cytotoxicity for human skin cancer *in vitro*

**DOI:** 10.1186/1423-0127-19-65

**Published:** 2012-07-17

**Authors:** Nahlah M Almansour, Elena Pirogova, Peter J Coloe, Irena Cosic, Taghrid S Istivan

**Affiliations:** 1 Biotechnology and Environmental Biology, School of Applied Sciences, Science Engineering and Health College, RMIT University, Bundoora, VIC, 3083, Australia; 2 School of Electrical and Computer Engineering, Science Engineering and Health College, RMIT University, Melbourne, VIC, 3000, Australia; 3 Health Innovations Research Institute, RMIT University, Melbourne, VIC, 3083, Australia

**Keywords:** RRM-MV, Myxoma virus, Bioactive peptide, Melanoma, Carcinoma

## Abstract

**Background:**

Cancer is an international health problem, and the search for effective treatments is still in progress. Peptide therapy is focused on the development of short peptides with strong tumoricidal activity and low toxicity. In this study, we investigated the efficacy of a myxoma virus peptide analogue (RRM-MV) as a candidate for skin cancer therapy. RRM-MV was designed using the Resonant Recognition Model (RRM) and its effect was examined on human skin cancer and normal human skin cells *in vitro*.

**Methods:**

Cell cultures were treated with various concentrations of the peptides at different incubation intervals. Cellular morphological changes (apoptosis and necrosis) were evaluated using confocal laser scanning microscopy. The cytotoxic effects of RRM-MV on human skin cancer and normal human skin cells were quantitatively determined by cytotoxicity and cell viability assays. The effect on human erythrocytes was also determined using quantitative hemolysis assay. DNA fragmentation assay was performed to detect early apoptotic events in treated cancer cells. Furthermore, to investigate the possible cell signalling pathway targeted by the peptides treatment, the levels of p-Akt expression in skin cancer and normal cells were detected by immunoblotting.

**Results:**

Our results indicate that RRM-MV has a dose-dependent toxic effect on cancer cells only up to 18 h. The immunoblotting results indicated that the RRM-MV slightly increased p-Akt expression in melanoma and carcinoma cells, but did not seem to affect p-Akt expression in normal skin cells.

**Conclusions:**

RRM-MV targets and lethally harms cancer cells and leaves normal cells unharmed. It is able to reduce the cancer cell viability, disrupting the LDH activity in cancer cells and can significantly affect cancer progression. Further investigation into other cell signalling pathways is needed in the process leading to the *in vivo* testing of this peptide to prove its safety as a possible effective treatment for skin cancer.

## Background

Cancer is one of the leading causes of death worldwide. Its occurrence, including in developed countries, is increasing [1]. Although current treatment regimens, such as chemotherapy, immunotherapy, radiation therapy and/or surgery, have considerably improved patients’ overall survival and quality of life, many cancers remain incurable and unmanageable. Therefore, there is a need to develop safe and effective anti-cancer agents [2].

Recently, new technologies have been tested successfully. These include oncolytic virotherapy, in which certain viruses selectively infect and kill cancerous cells, leaving normal cells unharmed *in vitro*[3-6] and in xenografted mice *in vivo*[7-9].

Myxoma virus (MV) is a rabbit-specific poxvirus pathogen that in the past few years has been intensely investigated as a potential oncolytic candidate [10-13]. MV is non-pathogenic in humans and has the benefit of being able to selectively target and terminate a wide range (about 70%) of human cancerous cells [14]. Furthermore, *in vitro* investigations into MV show its ability to infect immortalized baby green monkey kidney (BGMK) cells and some primary human dermal fibroblasts [14]. The mechanism of how MV selects and targets malignant cells is believed to be related to a unique tropism associated with the dysregulated intracellular signalling pathways, found in the majority of human cancers such as the Akt pathway [3]. Cell transformation and oncogenic activity is linked to the kinase activity of Akt. Hence, proliferation of tumor cells results from the dysregulation of Akt activity [15,16]. It has been reported that Akt needs to be activated via interaction with a viral ankyrin-repeat host range factor before MV can infect human cancer cells [17].

Akt, or protein kinase B (PKB), is a serine/threonine kinase that plays a crucial role in the regulation of many cellular processes, including apoptosis or programmed cell death, proliferation, angiogenesis and metabolism [13,18-20]. It is activated via the phosphorylation of its central components ser-473/474 and thr-308/309 [21]. Akt can be upregulated by a range of biological stimuli, including growth factors, protein phosphatase inhibitors and phosphatidylinositol 3-kinase (PI3K) [22].

The resonant recognition model (RRM) is a novel approach to *de novo* design of therapeutic peptides [23]. It is a physico-mathematical model used for analysing protein structure and function; predicting protein active binding sites and functional mutagenesis; and protein activation using the applied electromagnetic radiation (EMR) of the defined frequency [24,25]. The RRM concept is based on the finding that protein biological function is characterised by certain periodicities (frequencies) in the distribution of electron-free energy along the protein sequence. By utilizing this approach, it is possible to predict key amino acids that can contribute to protein activity and to design new peptides with the desired biological activity. It was suggested that these short, synthetic peptides will have a high tissue penetration and relatively low production cost, and can be easily modified for improved bioavailability [24,26].

The bioactive peptide “RRM-MV” was computationally designed by the RRM to mimic the bioactivity of selected MV proteins. It is a linear, short peptide (18 aa; MW 2.34 kDa), with an estimated half-life of 30 h in mammalian reticulocytes [27]. A non-bioactive peptide RRM-C (22 aa; MW 2.45 kDa) was also designed by RRM as a negative control, with an estimated half-life of 1.2 h in mammalian cells [28].

Our previous study [27] demonstrated that RRM-MV has a toxic effect on different types of mammalian tumor cell lines, and that this bioactive peptide has targeted and lethally harmed cancer cells, but left normal cells unharmed. In the current study, we have extended the evaluation of the toxic effect of RRM-MV and RRM-C on transformed human skin cancer and normal cell lines using qualitative and quantitative cellular viability, cytotoxicity and DNA fragmentations assays. We have also investigated the effect of these two peptides on Akt cell signalling pathway in human skin cancer and normal cell lines.

## Methods

### Cell cultures

The human malignant melanoma cell line (MM96L), human squamous cell carcinoma cell line (COLO-16), normal human epidermal melanocytes cell line (HEM) and normal human dermal fibroblast cell line (HDF) were obtained from the School of Medical Sciences, RMIT University, Australia. MM96L and COLO-16 cell lines were cultured in Roswell Park Memorial Institute medium (RPMI) 1640 (GIBCO, Australia) supplemented with 10% heat-inactivated fetal bovine serum (FBS) (Bovogen serumBiologicals, Australia). HEM cell line was grown in medium 254, supplemented with 1% human melanocyte growth supplement (HMGS) (Cascade Biologics, Australia). HDF cell line was propagated in medium 106 supplemented with 2% Low Serum Growth Supplement (LSGS) (GIBCO, Australia).

### RRM peptides preparation

The bioactive peptide RRM-MV and non-bioactive (negative control) peptide RRM-C were designed as previously described [27]. They were specifically designed with > 95% purity (AUSPEP, Melbourne, Australia). RRM-MV (MW 2.34 kDa) and RRM-C (MW 2.45 kDa) were freshly dissolved in either RPMI 1640, medium 254 or medium 106, according to the cell line used in the experiment. Peptide stocks were then prepared at the following concentrations: 50 ng/mL (*21.32 nM RRM-MV or 20.37 nM RRM-C*), 100 ng/mL (*42.6 nM RRM-MV or 40.7 nM RRM-C*), 200 ng/mL (*85.3 nM RRM-MV or 81.5 nM RRM-C*), 400 ng/mL (*170.5 nM RRM-MV or 162.9 nM RRM-C*), 800 ng/mL (*341 nM RRM-MV or 325.9 nM RRM-C*) and 1600 ng/mL (*682 nM RRM-MV or 651.7 nM RRM-C*).

### Apoptosis and necrosis assay

The effects of the RRM-designed peptides on cellular apoptosis and necrosis were determined by the apoptosis and necrosis assay. Cells were stained with the Annexin V-Alexa Fluor 488 (AF488) conjugate and Propidium Iodide (PI) as described in the manufacturer's protocol manual (Vybrant Apoptosis Assay kit II, Invitrogen, USA), with minor modifications. In brief, the cells were seeded at a density of 3 × 10^5^ cell per well in a 24-well plate and incubated overnight. Cells were treated with 100 ng/mL, 200 ng/mL and 400 ng/mL of RRM-MV or RRM-C, then incubated for 3 h or 18 h. After incubation, they were washed with cold phosphate-buffered saline (PBS). To each sample, 5 μL of AF488 and 1.5 μL of PI were added. These samples were then incubated at room temperature for 20 min before being washed twice and resuspended in a binding buffer (10 mM HEPES, 140 mM NaCl, 2.5 mM CaCl_2_ at pH 7.4). Stained cells were kept protected from light until they were examined by confocal laser scanning microscopy (CLSM). CLSM images were taken at 10, 20 and 40 × magnifications, with the pinhole aperture set at 1 using Nikon Eclipse Ti-E A1 laser-scanning confocal system (Nikon Instruments Inc, Japan). Images were analysed with the NIS-Element imaging software. All samples in duplicates were tested at least three times in duplicates.

### Lactate dehydrogenase (LDH) assay

The effect of the bioactive peptide RRM-MV on cell cytotoxicity was determined using the LDH assay. Cells were seeded at a density of 3 × 10^5^ cell per well in a 96-well plate and incubated overnight. They were then treated in triplicate with 400 ng/mL of RRM-MV or RRM-C and incubated for another 3 h. The LDH released from damaged cells was measured by Cytotoxicity Detection Kit (Roche Diagnostics, USA) according to the manufacturer’s instructions. The background control values were subtracted from each well and the mean percent treatment induced cytotoxicity for each cell line was calculated using the following equation: 100 × [(experimental value – low control)/(high control – low control)], where:

low control = mean absorbance from the untreated cells (spontaneous release of LDH) and high control = mean absorbance from lysis cells (maximum release of LDH) (positive control).

### MTT (3-(4,5-dimethylthiazol-2-yl)-2,5-diphenyl tetrazolium bromide) cell viability assay

The effect of the RRM-designed peptides on cell viability was determined using the MTT assay as described by Mosmann [29]. Briefly, cells were seeded at 3 × 10^5^ cells per well, in a 96-well plate and incubated overnight. Cells were then treated with 100 ng/mL, 200 ng/mL, 400 ng/mL, 600 ng/mL, 800 ng/mL, 1000 ng/mL, 1200 ng/mL, 1400 ng/mL and 1600 ng/mL of the RRM-designed peptide for 3 h incubation, or 25 ng/mL, 50 ng/mL, 100 ng/mL, 200 ng/mL, 400 ng/mL, 600 ng/mL and 800 ng/mL) for 18 h incubation. Followed by adding 25μL of the MTT solution (5 mg/mL; Sigma-Aldrich Company, St. Louis, MO), while cells were protected from light. After 4 h incubation, under standard conditions of 5% CO_2_ and 37 °C, the purple formazan product became visible. The precipitated formazan was dissolved by adding 100 μL dimethyl sulfoxide (DMSO) (Millipore, France) and placing it on a shaker for 5 minutes. The absorbance was read on ELISA plate reader (Thermo Electron Corporation, USA) at 595 nm. The blank values (medium) were subtracted from each well of the untreated and treated cells. The results were reported as percentage of cell death where the OD measure from untreated cells was considered to be 0% of cell death. The percentage of cell death was calculated as [1 – (OD 595 nm experiment/OD 595 nm untreated cells)] × 100.

### DNA fragmentation assay

DNA fragmentation assay was used to evaluate the effect of RRM-MV on DNA fragmentation in skin cancer cells. Cells were grown in a six-well plate at a density of 7 × 10^5^ cell per well and incubated overnight. MM96L and COLO-16 cells were incubated with 200 ng/mL and 400 ng/mL RRM-MV or RRM-C for 18 h. Following incubation, DNA was extracted using a Wizard® Genomic DNA Purification Kit (Promega, Australia) according to the manufacturer’s protocol. DNA concentration was quantified using a spectrophotometer at 260 nm and 280 nm. DNA was detected by agarose gel electrophoresis on a 1.5% agarose gel at 100 V for 1 h. Gels were stained with 3 μg/mL of ethidium bromide and visualized under ultraviolet light using the BioRad Gel Doc system.

### Immuno-blots for Akt Phosphorylation

Total Akt and p-Akt were detected by immuno-blotting to determine the effect of RRM-designed peptides on Akt pathway in human skin cancer and normal human skin cells.

### Antibodies and reagents

Rabbit monoclonal p-Akt (Ser-473) (D9E) XP antibody, rabbit monoclonal p-Akt (Thr308) (C31E5E) antibody, and rabbit polyclonal Akt (pan) (C67E7) primary antibody were all purchased from Cell Signaling Technology, Inc. (Beverly MA, USA). The alkaline phosphatase-conjugated goat anti-rabbit polyclonal secondary antibody was obtained from Sapphire Bioscience, Australia.

### Protein extraction

Cells were seeded at a density of 7 × 10^5^ cell per well in a six-well plate in complete growth medium with 10% FBS and incubated overnight. Cells were then treated with 400 ng/mL of RRM-designed peptide and incubated for 3 h. Cells were washed twice with cold PBS. After treatment, PhosphoSafe™ Extraction reagent (Novagen, USA) mixed with Protease Inhibitor Cocktails (Sigma-Aldrich, USA) was added and the plate was incubated for 10 min at room temperature. Cells were scraped and centrifuged at 16,000 x*g* for 5 min at 4 °C. The supernatants were collected and the protein concentration was quantified by the Bradford method as previously described [30] using bovine serum albumin (BSA) as a standard.

### Immuno-blot analysis

Equal amounts of protein (30 μg per lane) from each sample were resolved on 12.5% SDS-polyacrylamide gel electrophoresis using Mini-PROTEAN Tetra Cell electrophoresis system (Bio-Rad, USA). iBlot Dry Blotting System (Invitrogen, USA) was used to transfer the protein from the gel to nitrocellulose membrane. Western blotting was carried out as described [3] with some modifications. The immune bands were detected using 5-Bromo-4-chloro-3-indolyl phosphate toluidine salt/Nitro blue tetrazolium chloride (BCIP/NBT) substrate solution (Amresco, USA) with detection buffer (100 mM Tris, 100 Mm NaCl at pH 9.5) (v/v). Membranes were incubated until full colour development occurred. Reaction was stopped by washing in distilled water for 5 min prior to being dried.

### Hemolysis assay

The hemolytic effect of the RRM-designed peptides on human erythrocytes was carried out in a 96 well plate as described [31] with minor modifications. In brief, 5 mL fresh, whole blood was collected from an antecubital vein using a 21-gauge needle into EDTA tube. The blood was then diluted 1:5 with PBS and centrifuged at 1000 xg for 10 min. The erythrocytes pellet was resuspended in PBS to a final concentration of 3% (v/v). The erythrocytes suspension was treated with an equal volume of RRM-MV or RRM-C at the following concentrations: 50 ng/mL, 100 ng/mL, 200 ng/mL, 400 ng/mL, 600 ng/mL, 800 ng/mL, 1000 ng/mL, 1200 ng/mL, 1400 ng/mL and 1600 ng/mL. A completely lysed erythrocyte pellet was used as a positive control. The erythrocytes pellet was resuspended in sterile distilled water to a final concentration of 3% (v/v) for complete lysis. Following incubation at 37 °C overnight, the plate was centrifuged at 1000 xg for 10 min. The supernatants were collected and the OD at 595 nm was measured using ELISA plate reader (Thermo Electron Corporation, USA).

### Statistical analysis

Statistical analysis was performed using the Microsoft Office 2003 Excel software (Microsoft Corporation, Redmond Washington USA) and SPSS statistical software (version 18.0, SPSS, Inc., Chicago, IL, USA). The statistical significance of the differences between treated groups, controls groups and untreated groups were analyzed by one-way ANOVA and Tukey post hoc test. Experiments were performed at least three times independently. All values are expressed as mean ± standard errors. Results were considered significant at *P* < 0.05.

## Results

### Detection of cellular apoptosis and necrosis

**Figure 1:**
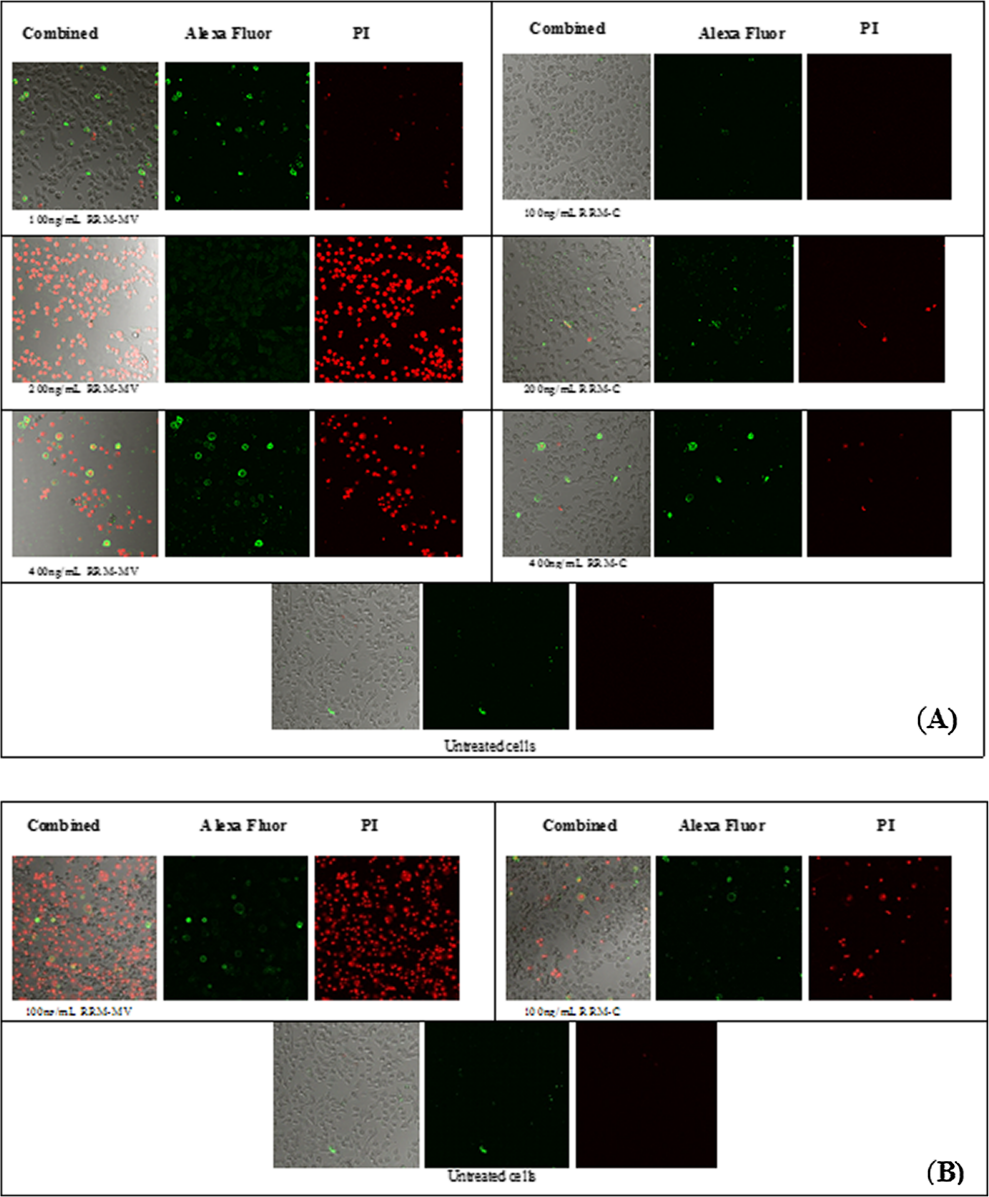
**CLSM micrographs of apoptosis and/or necrosis in malignant melanoma cell line (MM96L).** Cells were incubated for 3 h with 100 ng/mL, 200 ng/mL or 400 ng/mL of RRM-MV or RRM-C in (**A**); or with 100 ng/mL of RRM-MV or RRM-C for 18 h in (**B**). After incubation, cells were double stained with AF488 and PI. CLSM images show that RRM-MV induced cell apoptosis/necrosis effects. Prolonged incubation with RRM-MV caused stronger cytotoxic effects. AF488 channels show apoptotic cells labeled with green-fluorescent, and PI channels show necrotic cells labeled with red-fluorescent. CLSM images were taken with Nikon Eclipse Ti-E A1 laser-scanning confocal system, and analysed with the NIS-Element imaging software (100x magnification).

**Figure 2:**
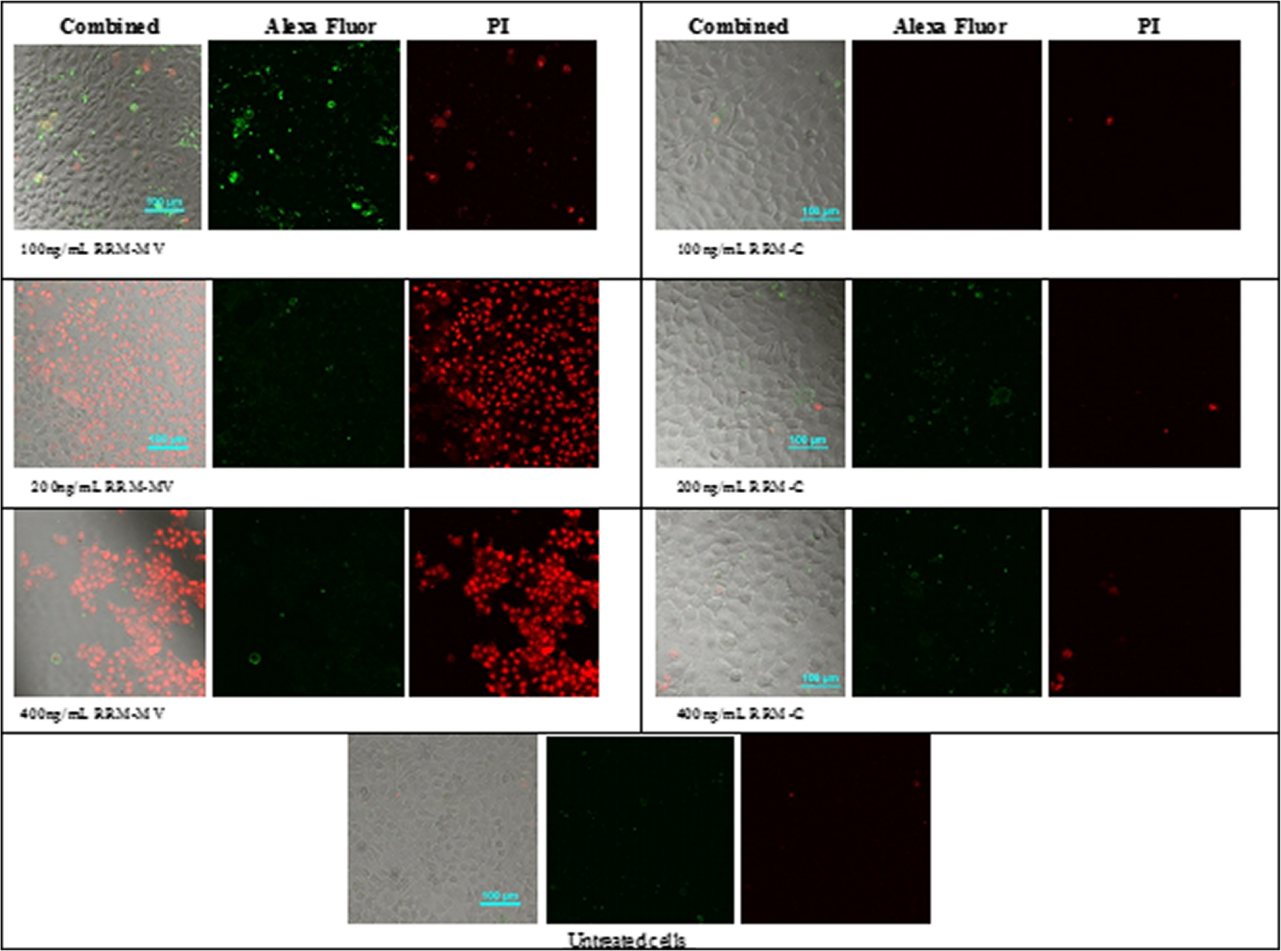
**CLSM images of apoptosis and/or necrosis in human squamous cell carcinoma (COLO-16) cell line.** Cells were incubated for 3 h with 100 ng/mL, 200 ng/mL or 400 ng/mL of RRM-MV or RRM-C. After incubation, cells were double stained with AF488 and PI. These CLSM images show that RRM-MV induced cell apoptosis/necrosis effects. AF488 channels show apoptotic cells labeled with green-fluorescent and PI channels show necrotic cells labeled with red-fluorescent (100x magnification).

The cytotoxic effects of RRM-MV and RRM-C were detected on the human cancer cell lines (MM96L and COLO-16) at different concentrations and different incubation times using CLSM. The characteristic morphological changes associated with apoptosis and/or necrosis were observed in these cell lines after RRM-MV treatment. Incubation of MM96L with 100 ng/mL of RRM-MV for 3 h induced slight apoptotic or necrotic effects which were not seen in RRM-C-treated and untreated cells (Figure 1A). Conversely, prolonged incubation of up to 18 h at this concentration caused significant apoptotic and necrotic effects in MM96L which were not seen in RRM-C-treated and untreated cells (Figure 1B). Similar effects were observed when the COLO-16 cell line was treated with 100 ng/mL of RRM-MV for 3 h (Figure 2) or for 18 h.

Treatment with higher concentrations (200 ng/mL and 400 ng/mL) of RRM-MV produced significant apoptotic and necrotic effects and detachment in MM96L and COLO-16 cell lines after only 3 h of incubation (Figure 1A and 2, respectively). Interestingly, no significant cytotoxic effects were detected when MM96L and COLO-16 cells were treated with similar concentrations of the negative control peptide RRM-C for 3 h and 18 h.

**Figure 3:**
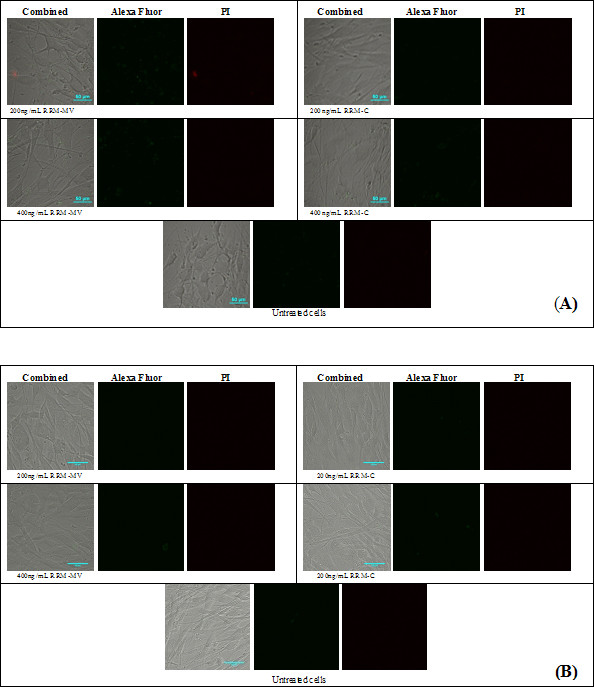
**CLSM images of normal human epidermal melanocytes (HEM) and normal human dermal fibroblast (HDF) cell lines following incubation with RRM-MV or RRM-C.** HEM cells in (**A**) and HDF cells in (**B**) were treated with 200 ng/mL or 400 ng/mL of either RRM-MV or RRM-C for 3 h, then double stained with AF488 and PI. No cytotoxic changes were detected in either cell line treated with RRM-MV.

Cellular apoptosis and/or necrosis were also assessed by CLSM in normal human skin cell lines (HEM and HDF). These cell lines were treated with 200 ng/mL and 400 ng/mL of RRM-MV or with similar concentrations of RRM-C. After 3 h of incubation, the CLSM micrographs indicated that neither apoptotic nor necrotic effects were observed in HEM and HDF cell lines treated with RRM-MV (Figure 3A and 3B, respectively). Taken together, no significant cytotoxic effects were observed on these cell lines after treatment either with RRM-MV or RRM-C.

### Treatment with RRM-MV induced lactate dehydrogenase (LDH) release

**Figure 4:**
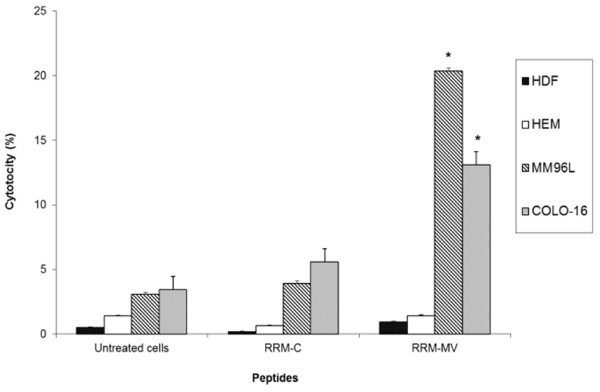
**Cytotoxic effects measured by LDH enzymatic activity in human skin cancer and normal human skin cells after treatment with RRM-MV or RRM-C.** LDH levels were measured following treatment with 400 ng/mL of RRM-MV or RRM-C for 3 h. RRM-MV produced high cellular LDH levels in cancer cells. Results are average of three independent experiments. Cytotoxicity values were analysed using one-way ANOVA and Tukey post hoc test. They showed a significant difference between RRM-MV-treated cells and untreated cells (p < 0.01). Significant values are indicated with an *.

Quantification of LDH activity released from damaged cells was measured using the LDH assay. The ability of the RRM-MV to induce LDH release from MM96L, COLO-16, HEM and HDF cell lines was investigated. Cells were incubated with 400 ng/mL of RRM-MV or RRM-C for 3 h. Data analysis of results revealed that the release of LDH from cells into the culture medium increased significantly after 3 h in RRM-MV-treated cancer cells compared with untreated cells. This suggested that treatment with 400 ng/mL of RRM-MV caused plasma membrane rupture and cellular damage in cancer cells. However, in normal cells, there was no significant difference in LDH release between RRM-MV-treated and untreated cells. Interestingly, RRM-C treatment did not induce significant LDH releases and was non-toxic for all tested cell lines (Figure 4). In total, the results indicated that the plasma membrane of the two normal cell lines remained intact during incubation with either the bioactive peptide RRM-MV or non-bioactive peptide RRM-C. Yet, RRM-MV treatment significantly induced cytotoxicity in cancer cells.

### RRM-MV induces cell death in dose and time-dependent manners in carcinoma and melanoma cell lines

**Figure 5:**
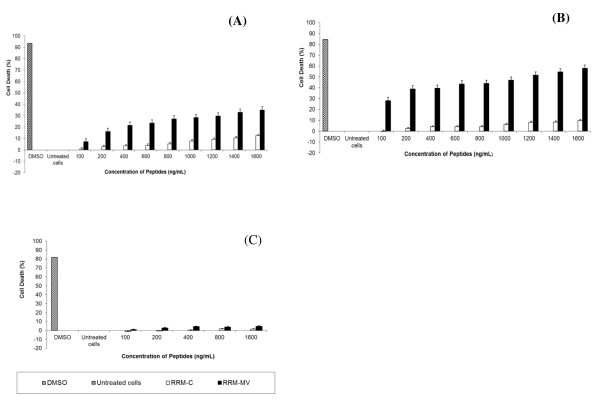
**Cellular viability of human skin cancer and normal human skin cell lines treated with RRM-designed peptides for 3 h measured by MTT assay.** MM96L cells in (**A**), COLO-16 cells in (**B**), and HDF cells in (**C**) were incubated with different concentrations of RRM-MV or RRM-C for 3 h. Cells treated with 90% DMSO were used as positive controls. Results are reported as percentage of cell death, where the optical density (OD_595nm_) value from untreated cells was set at 0% of cell death. The bar graphs show mean ± standard error of three independent experiments. Statistical significance of the differences between RRM-MV-treated cells and untreated cells were analysed using one-way ANOVA and Tukey post hoc test. The cellular viability values of RRM-MV-treated melanoma and carcinoma cells were significantly different from those of untreated melanoma and carcinoma cells (*P* < 0.001).

**Figure 6:**
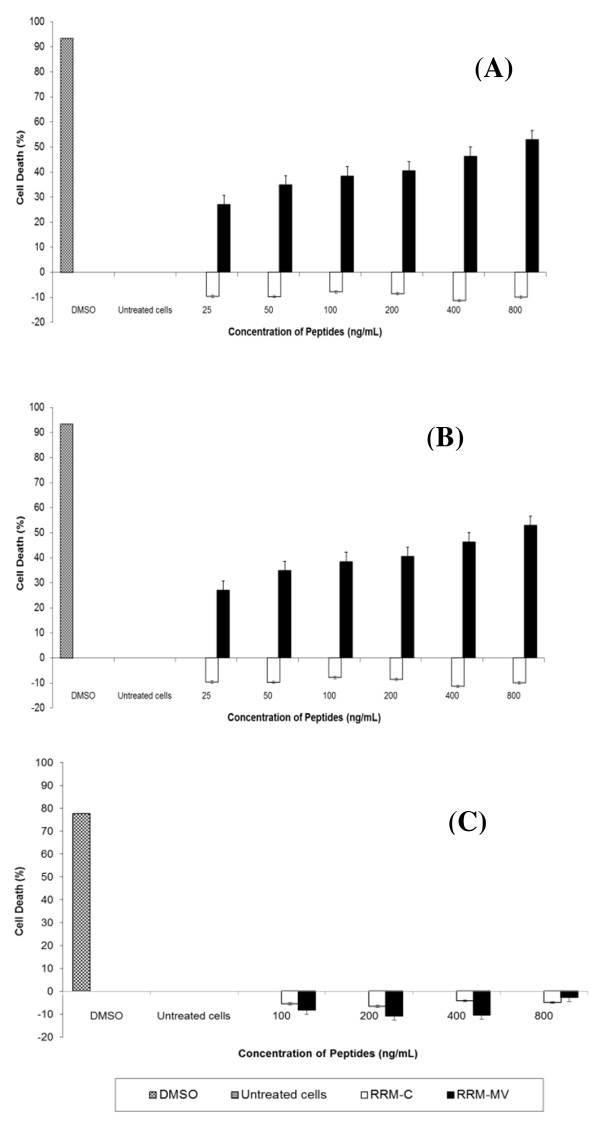
**Cellular viability of human skin cancer and normal human skin cell lines treated with RRM-designed peptides for 18 h measured by MTT assay.** MM96L cells in (**A**), COLO-16 cells in (**B**), and HDF cells in (**C**) were treated with different concentrations of RRM-MV or RRM-C for 18 h. Cells from each cell line were treated with 90% DMSO and used as positive controls. The effect of the peptides on cell viability was determined by MTT assay. Results are reported as percentage of cell death, where the optical density (OD) value from untreated cells was set at 0% of cell death. Bars indicate the mean ± standard error of three independent experiments. Statistical evaluation was performed by using one-way ANOVA and Tukey post hoc test. The RRM-MV-treated cancer cells were significantly different from untreated cells (*P* < 0.001).

The effects of different doses and incubation periods of RRM-MV or RRM-C on cell death were determined in MM96L and COLO-16 cell lines using the MTT cell viability assay. Cells were treated with different concentrations of peptides for 3 h or 18 h. Cancer cells were treated with (100 ng/mL, 200 ng/mL, 400 ng/mL, 600 ng/mL, 800 ng/mL, 1000 ng/mL, 1200 ng/mL, 1400 ng/mL and 1600 ng/mL) of either RRM-MV or RRM-C for 3 h. Data analysis of results indicated that cell death in MM96L and COLO16 increased noticeably with higher doses of RRM-MV compared with RRM-C-treated and untreated cells (p <0.001) in both cell lines. The maximum percentage of cell death was 34.92% and 57.98% at 1600 ng/mL of RRM-MV in MM96L and COLO-16 cell lines, respectively (Figure 5A and 5B). Additionally, incubation of cells with 25 ng/mL, 50 ng/mL, 100 ng/mL, 200 ng/mL, 400 ng/mL and 800 ng/mL RRM-MV for 18 h caused a statistically significant reduction in cell viability. At the highest peptide concentration (800 ng/mL RRM-MV), the percentage of cell death was 52.9% and 59.95% in MM96L and COLO-16 cell lines respectively (p <0.001 in MM96L and COLO16). Interestingly, RRM-C treatment for either 3 h or 18 h in both cell lines had negligible effects on cell viability even at the highest concentrations used (1600 ng/mL) (Figure 6A and 6B). Cytotoxic responses against RRM-MV treatment substantially increased in the cancer cells with increased doses. Hence, RRM-MV treatment decreased cell survival through the induction of cell death in dose- and time-dependent manners within 18 h. However, treatment of cancer cells with RRM-C displayed minimal effects on the tested cancer cell lines.

### The RRM-MV had no effect on cell viability in the normal human skin cell line (HDF)

HDF human skin cell line was incubated with RRM-MV or RRM-C 100 ng/mL, 200 ng/mL, 400 ng/mL, 800 ng/mL and 1600 ng/mL for 3 h, and 100 ng/mL, 200 ng/mL, 400 ng/mL and 800 ng/mL for 18 h. Toxic effects of these peptides were evaluated by MTT assay. There appeared to be no significant difference in HDF viability between RRM-MV-treated and untreated cells. No significant loss in viability was observed with RRM-C treatment (Figures 5C and 6C). Data analysis of results indicated that even the highest concentration of peptides treatment had negligible toxic effects on the tested normal human skin cells.

### RRM-MV induced DNA degradation in human skin cancer cell lines

**Figure 7:**
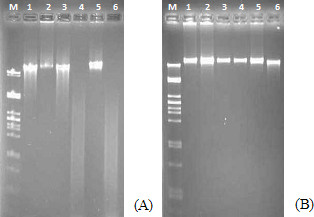
**Effect of RRM-MV treatment on the DNA of cancer cell lines.** MM96L cells in (**A**) and COLO16 cells in (**B**) were incubated with 200 and 400 ng/mL of RRM-C or RRM-MV for 18 h. DNA was extracted and analyzed by electrophoresis on 1.5% agarose gel with ethidium bromide staining. DNA samples are labeled as: (M) molecular weight marker, (1) untreated cells, (2) cells treated with DMSO as positive controls, (3) cells treated with 200 ng/mL RRM-C, (4) cells treated with 200 ng/mL RRM-MV, (5) cells treated with 400 ng/mL RRM-C, and (6) RRM-MV-treated cells. In MM96L cells, RRM-MV treatment produced DNA degradation, whereas in COLO16 cells, DNA degradation was not detected even with DMSO-treated samples. Results are representative of more than three independent experiments.

DNA fragmentation was analysed by agarose gel electrophoresis to detect apoptosis in human skin cancer cells. MM96L and COLO-16 cell lines were incubated with (200 ng/mL and 400 ng/mL) of RRM-MV or RRM-C for 18 h. Agarose gel analysis showed that RRM-MV treatment clearly degraded DNA at both peptide concentrations, in contrast with the untreated controls in MM96L cell line (Figure 7A, lane 4 and 6). Conversely, DNA degradation could not be observed in RRM-MV-treated COLO-16 cell line at similar concentrations within the same duration (Figure 7B, lane 4 and 6). The RRM-C treatment caused no changes in DNA patterns in MM96L and COLO-16 cell lines. Taken together, the RRM-MV treatment induced DNA degradation in melanoma cells which is the biochemical hallmark of apoptotic cell death, however, this degradation could not be observed in carcinoma cells.

### The effect of RRM-MV on the Akt signalling pathway

**Figure 8:**
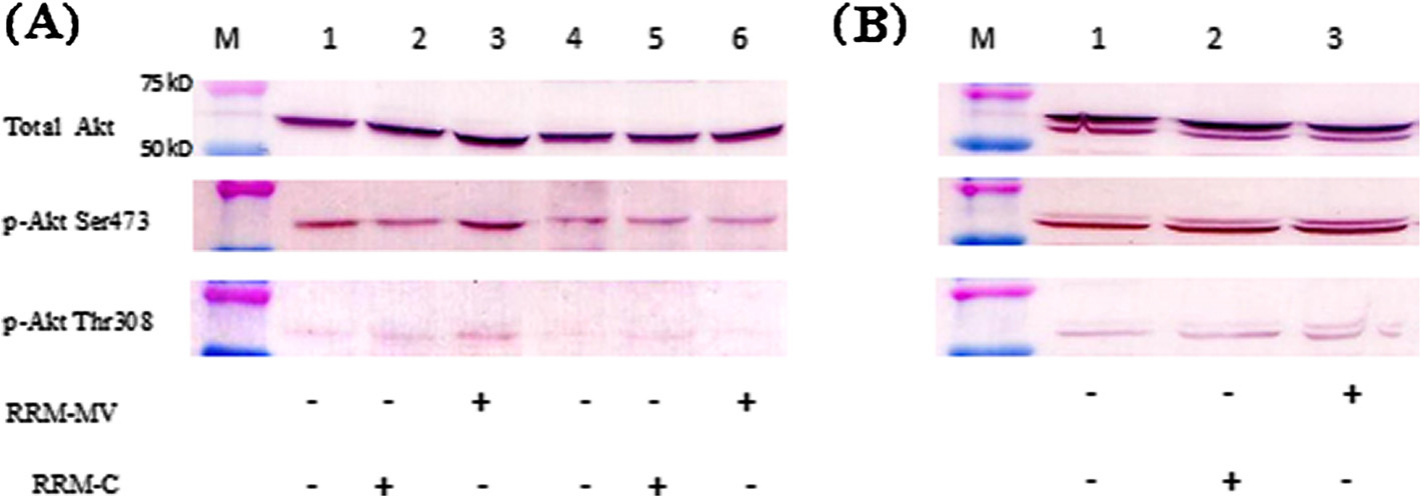
**The effect of RRM-designed peptides on cellular expression of p-Akt and total Akt.** Western blots for cancer cell lines MM96L and COLO-16 are presented in (A) and HDF cell line in (B) as indicated below the WB. Cell lysates were prepared and immune-blotted using p-Akt (Ser473), p-Akt (Thr308) and total Akt antibodies (around 60 kDa). The WBs show that RRM-MV treatment slightly affected p-Akt expression in the MM96L and COLO-16 cell lines, but did not affect the expression of p-Akt or total Akt in HDF cell line. These figures are derived from multiple immuno-blotting experiments.

The effect of RRM-MV or RRM-C treatment on Akt signalling pathways in MM96L, COLO-16 and HDF cell lines was investigated using immuno-blotting. All cell lines were incubated with RRM-MV or RRM-C for 3 h at the concentration of 400 ng/mL. Western blot results indicated that the immune band intensities of phospho-Akt at (Thr-308 and p-Akt Ser-473) were slightly increased in MM96L and COLO-16 cells after RRM-MV treatement (Figure 8A, lanes 3 and 6, respectively). However, there was also no difference in the intensity of the immune bands for p-Akt at (Thr-308 and p-Akt Ser-473) and total Akt in the HDF cell line after RRM-MV treatment (Figure 8B, lane 3). The RRM-C treatment did not seem to affect the intensity of the immune bands of p-Akt and total Akt in tested cell lines as compared with untreated cells (Figure 8A and B). Taken together, the RRM-MV treatment did not appear to significantly affect the expression of Akt in HDF cell lines; however, there was a slight increase in the expression of p-Akt in the RRM-MV-treated MM96L and COLO16 cell lines.

### Effect of RRM-designed peptides on human erythrocytes

**Figure 9:**
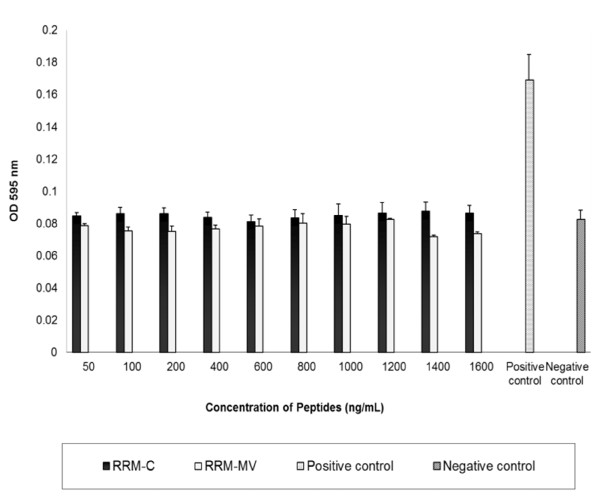
**The effect of the RRM-designed peptides on human erythrocytes.** Washed human erythrocytes in PBS were incubated for 18 h with the following concentrations of RRM-MVor RRM-C: 50 ng/mL; 100 ng/mL; 200 ng/mL; 400 ng/mL; 600 ng/mL; 800 ng/mL; 100 ng/mL; 1200 ng/mL; 1400 ng/mL; and1600 ng/mL. Human red blood cells were relatively resistant to RRM-MV and RRM-C treatment when incubated for up to 18 h, as cytolytic/hemolytic effects were not detected after treatment with both peptides. Data values are the average of three independent experiments in triplicates.

The toxicity of RRM-MV on human erythrocytes was tested using the hemolysis assay to evaluate the suitability of RRM-MV as a future therapeutic agent. Human erythrocytes were incubated with various concentrations of RRM-MV (50 ng/mL, 100 ng/mL, 200 ng/mL, 400 ng/mL, 600 ng/mL, 800 ng/mL, 1000 ng/mL, 1200 ng/mL, 1400 ng/mL and 1600 ng/mL) for 18 h. Similar concentrations of RRM-C were also tested on human erythrocytes for comparison. Results indicated that treatment of human erythrocytes with RRM-MV or RRM-C did not induce erythrocytes lysis at any of the concentrations used when compared with the complete erythrocyte lysis positive control (Figure 9). Thus, human erythrocytes appeared to be relatively resistant to RRM-MV and RRM-C treatments.

## Discussion

Peptide therapy has been applied in developing potential cancer therapeutics. These therapies focus on short peptide with strong tumoricidal activity and low toxicity [23,28,32,33]. We recently applied the RRM approach to design a short linear bioactive peptide (RRM-MV), mimicking the bioactivity of specific MV proteins. The RRM-MV has selectively potent antitumour cytotoxic effects on mouse melanoma *in vitro* in dose- and time-dependent manners [27]. We also used RRM to design the peptide analogue IL-12 to mimic the activity of mouse interlukin-12. This peptide produced cytotoxic effect on the B16F0 mouse melanoma cancer cells (B16F0) [33].

The current study examined and evaluated the suitability of RRM-MV as a candidate for human skin cancer therapy. The toxic effects of RRM-MV on the viability of human skin cancer and normal human skin cell lines were investigated. We found that RRM-MV induced apoptotic/necrotic effects in dose- and time-dependent manners in MM96L melanoma and COLO-16 carcinoma cell lines. We used AF488 and PI to detect apoptosis/necrosis in the plasma membrane. Early apoptotic cells were detected after 3 h of incubation with 200 ng/mL and 400 ng/mL RRM-MV. There were also many more apoptotic/necrotic cells to live cells in the RRM-MV-treated cancer cell lines when compared with the same ratio in RRM-C-treated and untreated cancer cell lines. When cancer cells were incubated with RRM-MV for a longer period (18 h), this bioactive peptide had a significant cytotoxic effect on these cells. In contrast, normal human skin cells were not affected by RRM-MV when incubated with it under similar conditions. Our findings suggest that the bioactive peptide RRM-MV can successfully induce apoptosis and necrosis in melanoma and carcinoma cells.

Moreover, this study showed that the level of LDH released from the RRM-MV-treated cancer cells was significantly higher (p < 0.01) than that released from untreated cells. The raise in LDH levels is known to be caused by an increased amount of dead cells or plasma membrane damage [29,34]. Our findings suggest that RRM-MV caused plasma membrane damage leading to a greater cytotoxic effect on cancer cells, but not on normal cells.

Increased LDH release was also confirmed by examining mitochondrial activity using MTT assay. Our data indicated that RRM-MV displayed significant cytotoxicity (*P* < 0.001) towards human skin cancer cells. Incubation with higher concentrations (up to 1600 ng/mL) of RRM-MV damaged cellular structures and made cells enter the necrotic death pathway, evident by the analysis of the cells’ morphology and increased LDH release with reduction in cell viability. On the other hand, in normal human skin cells neither RRM-MV nor RRM-C increased cell death within the investigated concentration range. This showed that the bioactive peptide has a negligible toxic effect on normal human skin cells, but high cytotoxicity towards cancer cells. In addition, the dose- and time-response study revealed that high concentration treatments (above 400 ng/mL) caused the greatest cytotoxic responses. This supported our previous findings that RRM-MV has cytotoxic effects in a variety of mammalian tumor cell lines [27,28]. Our data also showed that RRM-MV has minimal cytotoxicity on normal human skin cells even at high concentrations.

The apoptotic effect of RRM-MV was further confirmed by the DNA fragmentation assay. RRM-MV-treatment extensively degraded DNA in melanoma cells, suggesting that cells treated with RRM-MV underwent apoptosis. Normally, DNA degradation is considered a biochemical hallmark of apoptosis that involves cutting the inter-nucleosomal regions into small fragments of double-stranded DNA [35-37]. Thus, changing in the DNA characteristics after RRM-MV treatment confirmed cell death induction in melanoma cells. However, DNA degradation was not noticed in the RRM-MV-treated carcinoma cell line, although cellular cytotoxicity was confirmed by CLSM, LDH and MTT assays. This could be explained by the lack of endogenous DNases in this cell line [36]. Therefore, the potential of cells undergoing early apoptosis can not be eliminated in this cell line by the non-appearance of DNA degradation [38-40].

We also investigated if the Akt signalling pathway is targeted by RRM-MV as a possible cell death pathway in human skin cancer cells. It is believed that regulation of Akt activation is impaired in cancer cells [3], yet in some human cancer cells, it was shown that M-T5 protein of MV binds to p-Akt to regulate Akt signalling [41]. Data from this study indicated that the levels of p-Akt expression at Ser-473 and Thr-308 slightly increased in the RRM-MV-treated melanoma and carcinoma cells. Levels of p-Akt and total Akt were also unaffected by RRM-C treatment in both cancer cell lines. Likewise, levels of p-Akt and total Akt expression were unaffected after RRM-MV or RRM-C treatment in the normal human skin cell line. In our previous study we found that the Akt signalling pathway did not seem to be affected by RRM-MV treatment in the mouse melanoma cell line (B16F0) [27]. Wang *et al*. [3] reported that Akt phosphorylation can be activated by forming a complex between the viral protein (M-T5) and Akt in MV-infected permissive human cancer cell lines only.

Our study also showed that human erythrocytes were relatively resistant to haemolysis when treated with RRM-MV even at high concentrations. This supports the suitability of RRM-MV as a potential antitumor therapeutic agent.

## Conclusions

We have previously shown that RRM-MV produced toxic effects on the B16F0 mouse melanoma cell line. Here, we found that RRM-MV has a selective pro-apoptotic and cytotoxic activity on human melanoma and carcinoma cells without significantly affecting normal cells. Treatment of these cells with RRM-MV caused characteristic cellular morphology changes, apoptosis and/or necrosis, reduced cellular viability and induced cell membrane damage, while addition of the negative control peptide RRM-C produced no cytotoxic changes in these cells. Further research is currently undertaken to determine the exact mechanism in which cancer cells are affected by this bioactive peptide.

## Abbreviations

COLO-16, Human squamous cell carcinoma cell line; DNA, Deoxyribonucleic acid; HDF, Normal human dermal fibroblast cell line; HEM, Normal human epidermal melanocytes cell line; LDH, Lactate dehydrogenase; MM96L, Human malignant melanoma cell line; MV, Myxoma virus.

## Competing interests

The authors declare that they have no competing interests.

## Authors’ contribution

NMA designed, performed experiments and wrote the manuscript. TSI designed experiments and edited the manuscript. EP designed the peptides and approved the manuscript. PJC approved the manuscript and provided limited financial support. IC is the inventor of the RRM. All authors read and approved the final manuscript.
